# Genome-wide identification and characterization of BASIC PENTACYSTEINE transcription factors and their binding motifs in coconut palm

**DOI:** 10.3389/fpls.2024.1491139

**Published:** 2024-12-10

**Authors:** Zifen Lao, Jiali Mao, Runan Chen, Ran Xu, Zhuang Yang, Ying Wang, Junjie Zhou, Zhihua Mu, Hang Xu, Fengmei Li, Dongyi Huang, Yong Xiao, Jie Luo, Wei Xia

**Affiliations:** National Key Laboratory for Tropical Crop Breeding, School of Breeding and Multiplication (Sanya Institute of Breeding and Multiplication)/College of Tropical Agriculture and Forestry, Hainan University, Sanya, Hainan, China

**Keywords:** *CnBPC*, duplication, GA-motif, redundant, expression correlation

## Abstract

**Introduction:**

*BASIC PENTACYSTEINE* (*BPC*) is a small transcription factor family known for its role in various developmental processes in plants, particularly in binding GA motifs and regulating flower and seed development. However, research on the functional characteristics and target genes of *BPCs* in coconut (*Cocos nucifera*) is limited.

**Methods:**

In this study, we systematically characterized the gene structure, conserved protein domains, gene expansion, and target genes of *CnBPCs* in the coconut genome. We conducted yeast one-hybrid (Y1H) and dual-luciferase assay to explore gene interactions. We identified genes with the GA motif in their promoter regions and combined this information with a weighted gene co-expression network to identify the target genes of *CnBPCs*.

**Results:**

Eight *CnBPCs* were identified, including three Class I *CnBPCs* from triplication, four Class II *CnBPCs* (with *CnBPC6A* and *CnBPC6B* resulting from segmental duplication), and one Class III CnBPC (*CnBPC7*). Three conserved DNA-binding motifs were detected, exhibiting variation in certain sites. Widespread BPC gene expansion was detected in coconut and other plant species, while only three BPCs were found in the most basal extant flowering plant. Notably, 92% of protein-coding genes contained at least one GA motif, with the (GA)3 motif being most prevalent. Genes containing the GA motif that exhibit a high expression correlation with *CnBPCs*, tend to interact strongly with the corresponding *CnBPCs*. Additionally, promoters rich in the GA motif tend to interact with all members of *CnBPC*. The dual-luciferase assay showed that *CnBPCs* could activate or repress the transcriptional activities of promoters containing either (GA)3 or (GA)11 motif but with a bias toward certain genes. Furthermore, we constructed co-expressed networks identifying 426 genes with GA motifs as potential *CnBPC* targets.

**Discussion:**

Our findings suggest that *CnBPCs* may play significant roles in seed germination, flower development, and mesocarp development by interacting with genes such as *CnAG1*, *CnAG2*, *CnSTK*, *CnMFT*, and *CnCS*. This study characterized *CnBPCs*’ binding motif and possible target genes, laying a theoretical foundation to reveal *CnBPCs*’ function in flower and seed development.

## Introduction

The *BASIC PENTACYSTEINE* (BPC) family is a plant-specific transcription factor family that has five highly conserved cysteine residues in the basic zinc finger-like DNA-binding domain and assists in binding the GA-rich sequences (Berger and Dubreucq, 2012). BPCs play a critical role in diverse plant growth and development processes. Dysfunction of BPC can lead to pleiotropic developmental defects, such as dwarfism, small leaves, reduced lateral roots, aberrant ovules, and enlarged inflorescence meristem (Monfared et al., 2011; Simonini and Kater, 2014). BPCs are involved in the ovule development process as regulators of *INNER NO OUTER* (*INO*), a gene involved in ovule development (Meister et al., 2004), as well as regulators of the expression of the homeotic MADS-box gene *SEEDSTICK* (*STK*), to control the ovule identity (Kooiker et al., 2005). BPCs also act as regulators of key transcription factors to determine organ development, such as *LEAFY COTYLEDON 2* (*LEC2*) (Berger et al., 2011), *SHOOTMERISTEMLESS* (*STM*), *KNOX*, and *WUS* (Simonini and Kater, 2014). Moreover, increasing studies have indicated that *BPC*s displayed divergent functions in the regulation of crop traits, such as grain size and flowering time (Gong et al., 2018).

The BPC gene family is classified into three classes in *Arabidopsis*: Class I (*AtBPC1*–*3*), Class II (*AtBPC4*–*6*), and Class III (*AtBPC7*). Their function overlapped within classes and among classes for BPCs (Meister et al., 2004). The BPC proteins act redundantly to regulate plant development, as the higher-order bpc mutants display a variety of reproductive and vegetative defects, while single and double mutants display no or less severe phenotypes (Santi et al., 2003; Kooiker et al., 2005; Monfared et al., 2011; Berger and Dubreucq, 2012). Severe morphological phenotypes occur in higher-order mutants between members of Classes I and II (Monfared et al., 2011). Research on the promoter of *AtSTK* indicated, in addition to the Class I BPC proteins, that other BPC factors act redundantly in the regulation of *AtSTK* (Simonini et al., 2012). Analyzing and comparing the *BPC* expression pattern and target sequence bias would help explain why functional redundancy occurs.

The BPC genes identified in different plant species were characterized to bind dinucleotide repeats: the *GAGA BINDING PROTEIN* (GBP) of soybean specifically binds a (GA)_9_ repeat sequence (Sangwan and O’Brian, 2002), the *BARLEY B RECOMBINANT* (BBR) factor binds (GA)_8_ sequences *in vitro* (Santi et al., 2003), and the *Arabidopsis* BPC proteins specifically recognize (GA)_6_ and (GA)_9_ repeats *in vitro* and *in vivo* (Meister et al., 2004; Kooiker et al., 2005; Simonini et al., 2012). In *Drosophila*, at least two GA repeats are necessary for GAF recruitment, and 2.5 or 3 repeats appear to be optimal (van Steensel et al., 2003). Meanwhile, GAF binds with approximately the same frequency in either orientation relative to the direction of transcription (van Steensel et al., 2003). GA dinucleotide repeats were the conserved target motifs for BPC genes, while they are widespread in genome sequence and frequently detected within core promoter sequences (Yamamoto et al., 2009). There are more than 12,000 *Arabidopsis* genes containing more than one GA-rich stretch within their regulatory regions (Santi et al., 2003; Hecker et al., 2015). Screening and identifying the most likely targets of BPCs will provide a foundation for gaining deeper insights into their functions in various developmental processes.

Coconut (*Cocos nucifera*, 2n = 32), a member of the monocotyledonous Arecaceae (Palmaceae) family, is an important crop and landscape plant in tropical areas. Coconut is characterized by its large fruit and unique liquid endosperm-coconut water, which are consumed as food and water. In addition, it has various uses such as fiber, building materials, charcoal, and oil, and it is widely acknowledged as a tree of life. In a recent study, high-quality dwarf and tall coconut genomes were obtained, and key agricultural traits were genetically analyzed (Wang et al., 2021; Yang et al., 2021). The completion of genome sequencing for more species has enabled many genes to be identified and characterized. However, comprehensive analyses of BPC genes in coconut and other plant species are still limited. In the present study, we identified BPC family members in the coconut genome and divided them into three classes in comparison with *AtBPC*s. These classes were Class I (*CnBPC1* to *CnBPC3*), Class II (*CnBPC4*, *CnBPC5*, and *CnBPC6A*/*6B*), and *CnBPC7* in Class III. Subsequently, we analyzed their gene structures, and we subsequently analyzed conserved motifs. Furthermore, we analyzed the evolutionary characteristics of the plants by identifying and comparing homologous segments among 15 plant species. The transient expression assay indicated that all *CnBPC* proteins were localized in the nucleus. The GA motif-containing genes were analyzed in the coconut genome, and the interaction between CnBPC proteins and promoters of *CnAG1*, *CnAG2*, *CnSTK*, *CnMFT*, and *CnCS* was validated. Dual-luciferase assay indicated that CnBPCs could interact with the (TC)_3_ and (TC)_11_ motifs. Based on co-expression network construction and the GA motif features, 426 genes were identified as targets of CnBPCs. This work provides a basis for exploring the potential target genes of CnBPC genes and will lay a theoretical foundation for analyzing the function of *CnBPC*s in coconut flower and seed development.

## Materials and methods

### Data sources and sequence retrieval

The coconut (Cnu, Arecaceae) genome sequence, gene protein sequences, and the transcriptome datasets used in this study were generated from our previous research (Wang et al., 2021). Additionally, the genome sequence, gene model information, and gene protein sequences of the other 14 species—*Amborella trichopoda* (Atr, Amborellaceae), *Daucus carota* (Dca, Daucinae), *Solanum tuberosum* (Stu, Solanoideae), *Vitis vinifera* (Vvi, Vitaceae), *Malus domestica* (Mdo, Rosaceae), *Citrus sinensis* (Csi, Rutaceae), *Arabidopsis thaliana* (Ath, Brassicaceae), *Dioscorea alata* (Dal, Dioscoreaceae), *Phoenix dactylifera* (Pda, Arecaceae), *Elaeis guineensis* (Egu, Arecaceae), *Musa acuminata* (Mac, Musaceae), *Ananas comosus* (Aco, Bromeliaceae), *Brachypodium distachyon* (Bdi, Poaceae), and *Oryza sativa* (Osa, Poaceae)—were retrieved from the Phytozome website (http://www.phytozome.net/) and the National Center for Biotechnology Information (NCBI) website (https://www.ncbi.nlm.nih.gov/). The 14 species included one basal angiosperm species (Atr), six eudicots (Dca, Stu, Vvi, Mdo, Sci, and Ath), and eight monocot species (Dal, Egu, Pda, Cnu, Mac, Aco, Bdi, and Osa), with three characters contained in the parentheses to represent each species.

### Genome-wide identification of *BPC* genes

To identify *BPC* gene members, the hidden Markov model (HMMER) profile of the SRF-TF domain (Pfam accession: PF00319) was obtained from the Pfam database (http://pfam.xfam.org/) and used as a query to search against proteins of coconut and other 14 species described above. To classify the identified *BPC* genes into specific gene subfamilies, multiple sequence alignments were performed on the amino acid sequences using ClustalW with default parameters. *BPC* genes from coconut and 13 other species (excluding *Arabidopsis*) were then subjected to Basic Local Alignment Search Tool (BLAST) searches against *AtBPC*s to identify the best homologous hits. The BLAST results were also utilized to assign *BPC* subfamilies. The gene list and subfamily information for the 15 species are included in **Supplementary Table S1**.

### Evolutionary analysis of *BPC* genes

Multiple sequence alignments for *CnBPC*s and *AtBPC*s were applied to construct a phylogenetic tree using MEGA 7.0 with 1,000 bootstrap replicates (Kumar et al., 2016). A phylogenetic tree of coconut palm and 14 other species was also constructed in the context of angiosperms. The phylogeny was inferred using RAxML v8 with the PROTGAMMAJTT model, 1,000 bootstrap replicates, and 157 single-copy genes (Stamatakis, 2014).

### Identification of homologous segments with *BPC* genes between species

All protein-coding genes from coconut and the 14 species were aligned using BLAST against each other’s protein-coding gene databases with a cutoff of 1e−5. The BLAST results were processed using the software MCScanX to determine homologous chromosomal regions within coconut palm and between species, which contain *BPC* genes (Wang et al., 2012). All protein-coding genes were subjected to BLAST against themselves, and then BLAST results were processed using the software MCScanX to determine paralogous chromosomal regions. Duplicated gene pairs of *CnBPC*s in paralogous genomic segments were identified based on the following three criteria: a) the alignment covered >80% of the longer gene, b) the aligned region had an identity >80%, and c) only one duplication event was counted for the tightly linked genes.

### GA motif analysis in gene promoter region

The 2-kb upstream sequences starting from the start code of all protein-coding gene models were extracted using the Basic Local Alignment Search Tool. The start code location of each *CnBPC* gene was based on gene model information released in our previous research (Wang et al., 2021). Dinucleotides of GA/TC/CT/AG in the putative promoter region of coconut genes were analyzed using the software MISA (Beier et al., 2017).

### Transcriptome datasets and WGCNA

The RNA-seq Sequence Read Archives (SRAs) were created in our previous research, with the accession number CRA004778, which are publicly accessible at https://ngdc.cncb.ac.cn/gsa. The transcriptome dataset includes RNA-seq data for five types of tissues—leaf, flower, stem, endosperm, and mesocarp. Hisat2 was used to map reads to the coconut genome, and Fragments Per Kilobase Million (FPKM) values were calculated using StringTie. For the target genes *CnAG1*, *CnAG2*, *CnSTK*, *CnMFT*, and *CnCS*, their expression correlation with CnBPCs was calculated using Pearson’s correlation coefficient (PCC). The significance test of the PCC value was performed using *t*-test (cor.test in R).

Co-expression modules were generated based on the above coconut transcriptomes. The Reads Per Kilobase Million (RPKM) values were processed to remove genes showing low expression (RPKM ≤ 1 in all samples). The Weighted Gene Co-Expression Network Analysis (WGCNA) package installed in the R environment was used to identify co-expression modules for selected genes with min–max normalized and log2-transformed FPKM values. Modules were defined as clusters of highly interconnected genes, and genes within the same cluster had high correlation coefficients with each other. The gene modules were visualized using Cytoscape.

### Transient expression of *CnBPC* genes in tobacco epidermal cells for subcellular localization

The total RNAs were extracted from the mixed tissues containing leave, flower, endosperm, and mesocarp tissues using the AFTMag Quick Complex Plant RNA Extraction Kit (RK30181, ABclonal, Wuhan, China). The complementary DNAs (cDNAs) were prepared using the ABScript Neo RT MasterMk kit (RK20433, ABclonal, China). The full-length coding sequences of *CnBPC* were amplified using primers listed in **Supplementary Table S2**. *CnBPC6B* was excluded from further analysis because of low expression and no amplification product. The Uniclone One Step Seamless Cloning Kit (Genesand Biotech Company, Beijing, China) was used to construct the OE-expression *CnBPC* vector pc1300-35S-CnBPC-eGFP. The pc1300-35S-eGFP plasmid was linearized by digesting with *Sal*I and *Kpn*I, while *CnBPC* genes were amplified with primers containing homologous sequence fragments adjacent to the *Sal*I and *Kpn*I sites. Homologous recombination was used to link the *CnBPC* genes to the linear pc1300-35S-eGFP plasmid.

Agrobacterial cultures of 35S::CnBPC_(1-7)_:eGFP and 35S::OsGhd7:RFP (as positive control for nuclear localization) fusion constructs were pelleted and resuspended in the infiltration media. Empty vectors pc1300-35S-eGFP and pc1300-35S-RFP were used as negative control. Using a needleless syringe, the resuspended solution (OD_600_ = 1.0) was infiltrated into the abaxial surface of fully expanded tobacco leaves of 4-week-old plants. Green fluorescent protein (GFP) signals were detected at time intervals of 48–72 hours post-infiltration using a confocal microscope (LMS980, Zeiss, Oberkochen, Germany).

### Yeast one- and two-hybrid assays

Yeast one-hybrid (Y1H) assays were performed using the Y187-pHis2-bait system according to the Yeast Protocols Handbook (PT3024-1, Clontech, Mountain View, CA, USA). In brief, different fragments of the promoters containing the GA motif were cloned into the pHis2 vector and transformed in the Y187 strain. Self-autoactivation of the GA motif-pro-His2 was tested on SD/-Trp-His plates, and the minimum inhibitory concentrations for each bait strain were determined while containing different concentrations of 3-amino-1,2,4-triazole (3-AT). The full-length coding sequence of *CnBPC_(1-7)_* was inserted into the pGADT7 vector as the prey vector. *CnBPC_(1-7)_-pGADT7* was transformed into bait-specific reporter strains according to the method described in the manufacturer’s protocol. The transformed yeast cells were spread onto SD/-Trp-His-Leu/3-AT plates and cultured at 30°C for 3 days.

The full-length coding sequences of *CnBPC1*, *CnBPC5*, and *CnBPC7* were inserted into the pGBKT7 vector as the bait vector. Yeast two-hybrid (Y2H) assays were performed in the yeast strain AH109. For yeast transformation, yeast competent cells (AH109) were prepared according to the manufacturer’s instructions. Self-autoactivation of the CnBPC-BD was tested on SD/-TH plates. *CnBPC-pGBKY7* and *CnBPC_(1-7)_-pGADT7* constructs were co-transformed in AH109 competent cells. Cell growth of co-transformants was observed on nutrient medium lacking Leu and Trp (SD/-Leu/-Trp) and medium deficient in Ade, His, Leu, and Trp (SD/-Ade/-His/-Leu/-Trp) at 30°C for 3 days.

### Dual-luciferase transient expression assay

Dual-luciferase reporter gene assays were performed to verify the interaction between *CnBPC* and promoter sequence of *MFT* (MFTpro) and GA motifs (TC)_3_ and (TC)_11_. The full-length coding sequences of seven *CnBPC*s were amplified and linked to the pc1300-35S vector by homologous recombination described above. The promoter fragment of *MFT* and GA motifs were linked to the double-reporter vector *pGreenII 0800-LUC* reporter vector (*pLUC*), which contains a GAL4-LUC and an internal control REN. The *pLUC* vector was digested with *Kpn*I and *Hin*dIII (NEB, Ipswich, MA, USA). The GA motif sequence primers were paired with a PCR cycle (95°C for 3 min, 65°C for 10 min, 37°C for 10 min, and 12°C for 2 min) and then linked to the digested *pGreenII 0800-LUC* reporter vector with DNA T4 ligase (NEB). All primers utilized for vector construction can be found in **Supplementary Table S2**.

The recombinant plasmids were transformed into *Agrobacterium tumefaciens* GV3101 (Psoup-p19) (AC1003, Weidi, Jiangsu, China). Subsequently, the transformed *A. tumefaciens* was infiltrated into tobacco leaves using a needleless syringe (1 mL). After 48 h post-infiltration, the leaves were sampled, quickly frozen using liquid nitrogen, and ground into power. To determine the firefly luciferase (Luc) to *Renilla* luciferase (Ren) activity, the Dual-Luciferase Assay Kit (Promega, Madison, WI, USA) and the Infinite M200pro Full-Wavelength Multifunctional Enzyme Labeling Instrument (Tecan, Zürich, Switzerland) were utilized. The remaining infiltrated leaves from the same tobacco plants were subjected to chemiluminescence imaging. The imaging process was carried out using a live plant imaging system (PlantView100, Biolight, Zhuhai, China). Three biological replicates were established for each dual-luciferase assay. Each experiment was repeated three times.

## Results

### Gene characteristics of the BPC gene family (*CnBPC*s) in coconut palm

To identify the *BPC* genes in coconut, a hidden Markov model (HMMER) search against the coconut genome protein database was conducted using the GAGA_bind domain (PF06217) as a seed. The conserved BPC domains were detected by verifying sequences in the Pfam databases. Eight CnBPC proteins with complete domains were obtained and designated according to the best hits of *AtBPC* homologs (**Supplementary Table S1**). Therefore, *CnBPC1* to *CnBPC3*, *CnBPC4* to *CnBPC6A*/*B*, and *CnBPC7* belong to *BPC* classes I, II, and III, respectively.

**Figure 1:**
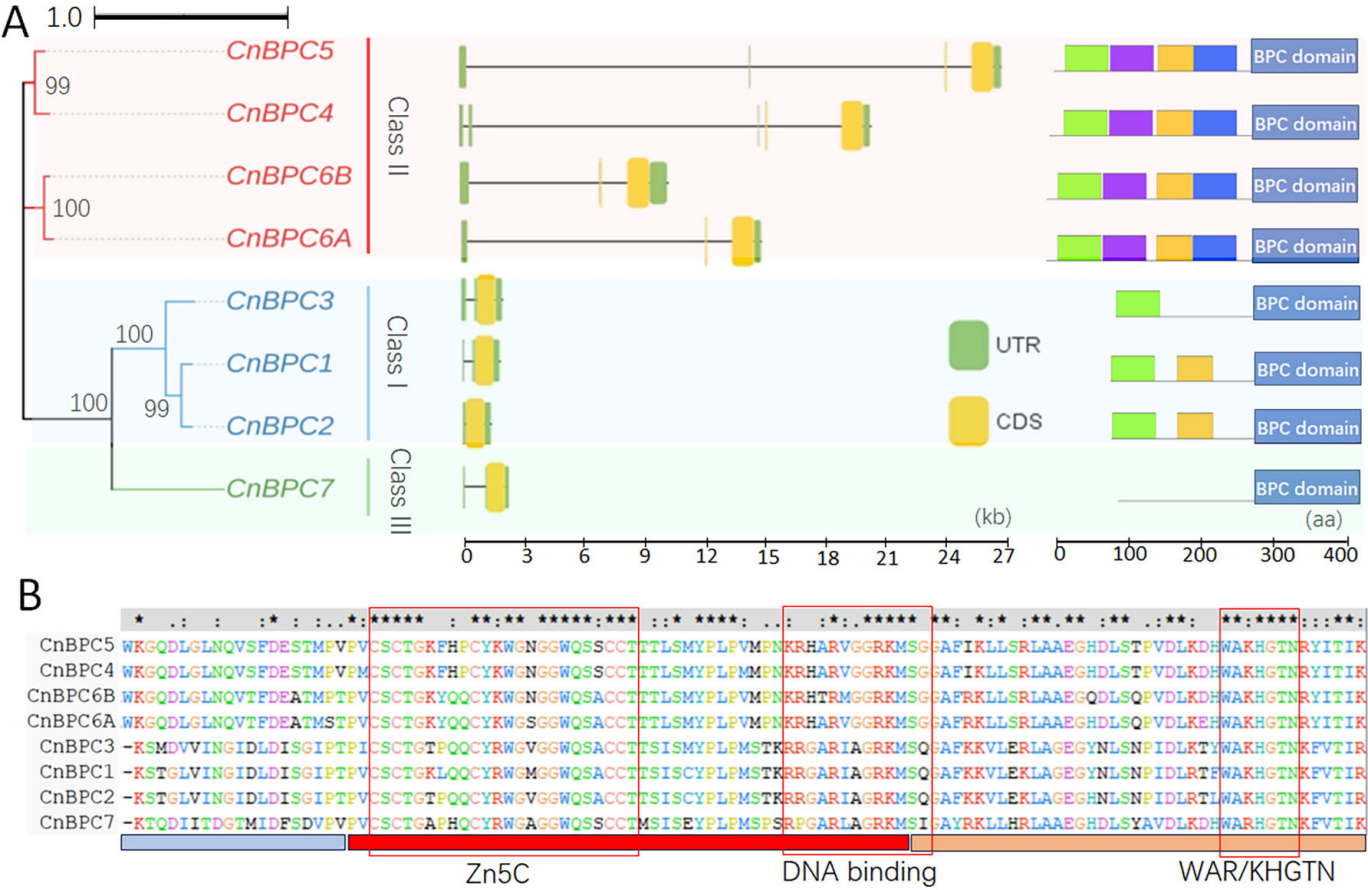
The phylogenetic tree, gene structure, and protein conserved domain of CnBPCs. **(A)** The phylogenetic tree of *CnBPC*s was constructed by MEGA 7.0 using the neighbor-joining method (left), as well as the gene structure (middle) and conserved protein motif (right) of the corresponding *CnBPC*s. The percentage of replicate trees in which the associated taxa clustered together in the bootstrap test (1,000 replicates). The evolutionary distances were computed using the Poisson correction method and are in the units of the number of amino acid substitutions per site. The gene structures are displayed using the software GSDS2.0. The conserved protein motif analysis was conducted using the software MEME online. **(B)** The amino sequence characteristics of BPC domain conserved between CnBPC proteins. Red box for the DNA-binding domain was derived from the PredictProtein online analysis (https://predictprotein.org/). WAR/KHGTN motif was required for DNA binding in *Arabidopsis* (Theune et al., 2019).

In the phylogenetic tree of *CnBPC*s, Class I and Class III showed a closer evolutionary relationship than Class II, including higher similarity in gene structure and conserved protein domains (**Figure 1A**). *CnBPC*s in Classes I and III only had introns in the 5′ untranslated region (5′UTR) for some members, while Class II members had introns in both 5′UTR and coding regions (**Figure 1A**, middle). As for the conserved protein motif detected by the MEME software, there were four conserved protein motifs in Class II but degraded corresponding motifs in Classes I and III (**Figure 1A**, right). All CnBPC proteins had a conserved BPC domain in the C-terminal, which is composed of three motifs with varying degrees of conservation (**Figures 1A**, right, **B**). The BPC domain was considered to have DNA-binding capacity, and the WAR/KHGTN motif was required for this function (Theune et al., 2019). In the CnBPC proteins, the putative DNA-binding region is characterized by the Zn5C motif, which consists of five conserved cysteine residues. This region contains 17 conserved amino acids, while the majority of the remaining residues exhibit high variability, even among gene members within the same class. Another DNA binding-related motif—RRGARIAGRKMSQ—was predicted *in silico*. This motif had six conserved amino acids and was conserved within BPC classes. The third DNA-binding motif (WAR/KHGTN) validated in *Arabidopsis* was very conserved in the seven CnBPC proteins except for the CnBPC7, which had one amino acid variation.

### *BPC* genes were highly conserved among plant species

**Figure 2:**
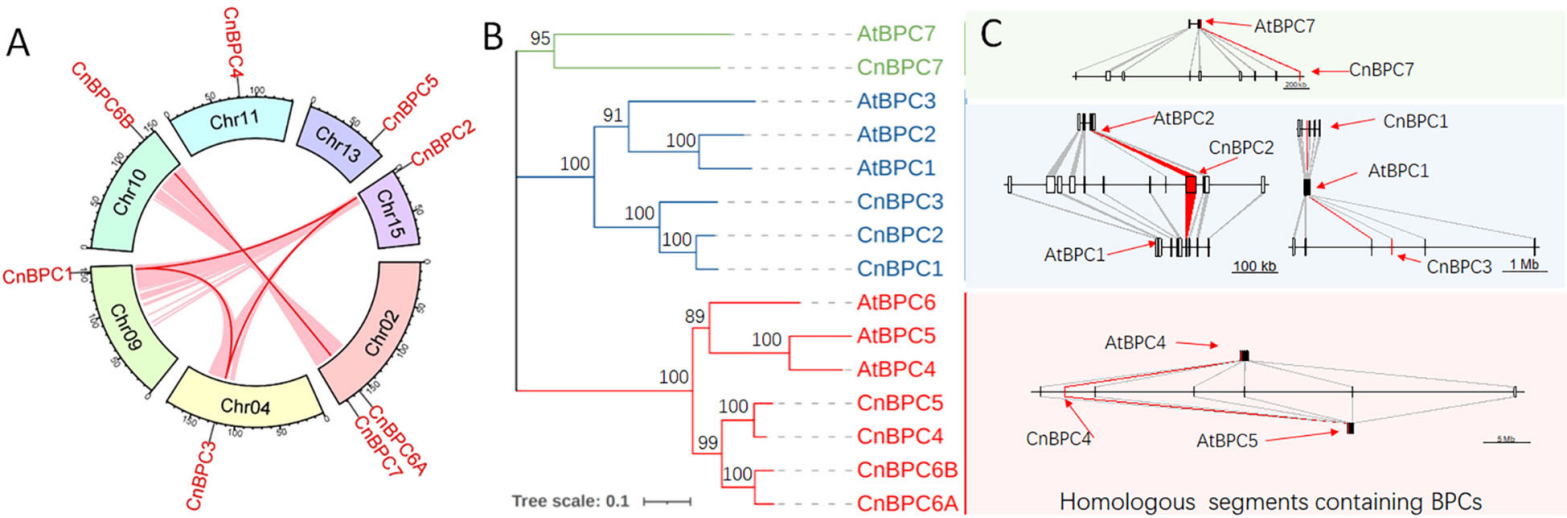
*CnBPC* gene members in coconut palm and comparison with *AtBPC*s. **(A)** The genomic locations of *CnBPC1* to *CnBPC7*, along with the duplicated genomic segments containing *CnBPC*s deduced from MCScanX analysis. **(B)** Phylogenetic tree of *CnBPC*s and *AtBPC*s was constructed by MEGA 7.0 using the neighbor-joining method with the same detail as in **Figure 1**. **(C)** Homologous genomic segments containing *CnBPC*s and *AtBPC*s, which were identified through MCScanX analysis.

Investigation into the gene expansion of *CnBPC*s revealed that genomic duplication was the primary driver behind the increase in gene members (**Figure 2A**). Although there were only eight gene members in total, segmental triplication contributed to the expansion of gene members in Class I. Additionally, segmental duplication led to the formation of a duplicated gene pair—*CnBPC6A* and *CnBPC6B*.

The comparison analysis of *CnBPC*s and *AtBPC*s indicated that the *BPC* genes from coconut and *Arabidopsis* correspond closely to one another, preserving conserved collinear segments. The phylogenetic tree constructed using *CnBPC*s and *AtBPC*s demonstrated that the gene members in the two species could be clustered into the three classes. The genes from each species clustered together within each class, indicating that the genes from *Arabidopsis* and coconut in each class share a common origin but have independently expanded in their respective species (**Figure 2B**). Furthermore, the identification of conserved syntenic regions of *BPC*s between *Arabidopsis* and coconut supports the notion that each *BPC* class originated from the corresponding genomic segments of a common ancestor (**Figure 2C**). All *CnBPC*s in Class I, which expanded through segmental triplication, are situated in syntenic regions with *AtBPC1* and *AtBPC2*. In Class II, *CnBPC4* is found in a syntenic chromosomal region alongside *Arabidopsis AtBPC4* and *CnBPC5*. Additionally, *CnBPC7* and *AtBPC7* in Class III are also located in collinear genomic blocks with each other.

**Figure 3:**
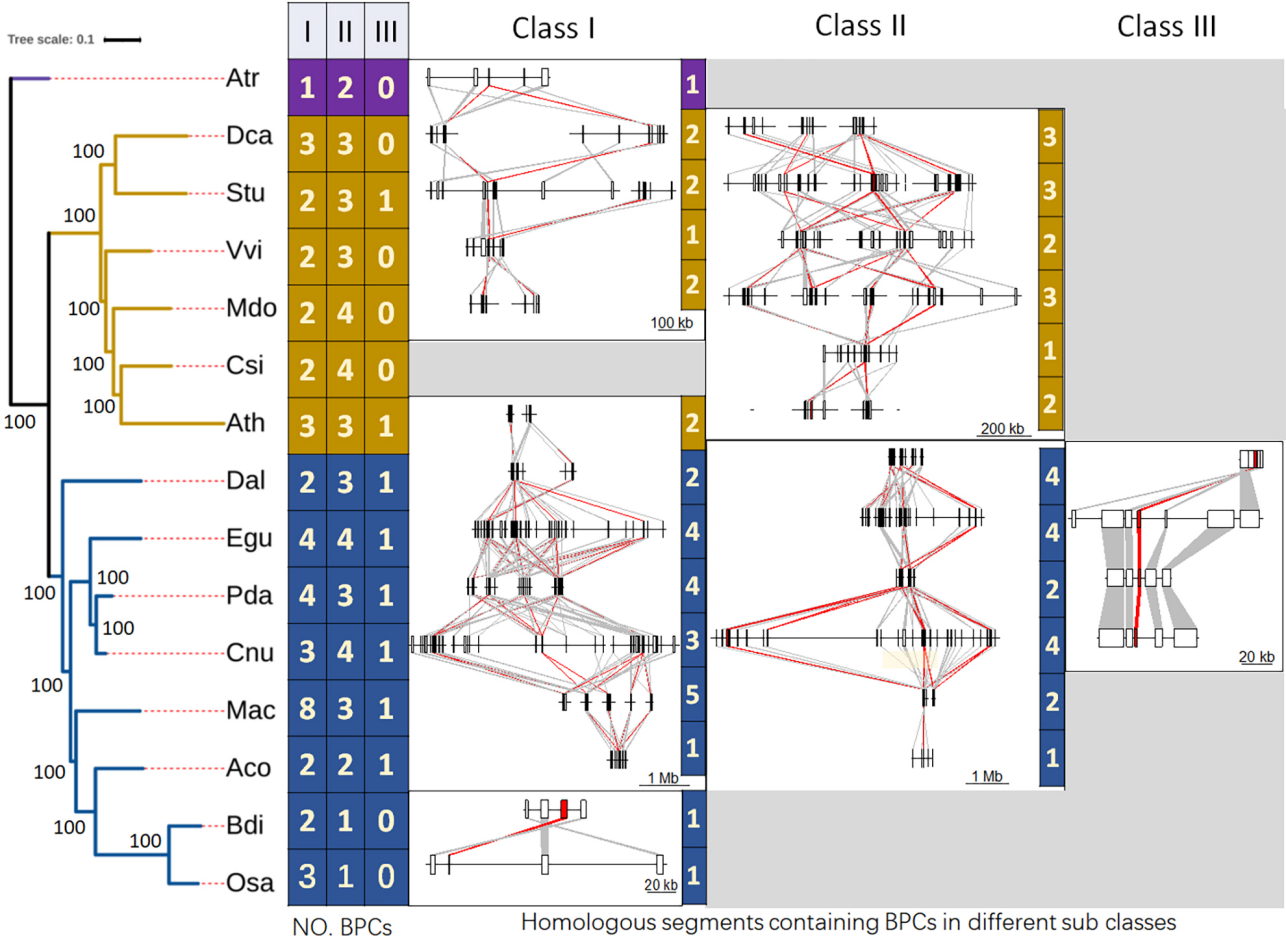
The phylogenetic tree, CnBPC gene numbers of coconut and 14 other species in the context of angiosperms, and homologous genomic segments between close species. The maximum likelihood (ML) phylogenetic tree of 15 species was constructed using 140 single-copy genes detected by orthfinder. The number of BPCs was calculated based on the BPC identified and class division in each species according to the method used for coconut. The homologous genomic segments for closely related species on the phylogenetic tree were identified by MCScanX analysis, with the number of the *BPC*s contained in homologous segments on the corresponding right box. The information of three characters representing the species is listed in the Materials and Methods section. Three-letter abbreviations were used to represent each species: *Amborella trichopoda* (Atr), *Daucus carota* (Dca), *Solanum tuberosum* (Stu), *Vitis vinifera* (Vvi), *Malus domestica* (Mdo), *Citrus sinensis* (Csi), *Arabidopsis thaliana* (Ath), *Dioscorea alata* (Dal), *Phoenix dactylifera* (Pda), *Elaeis guineensis* (Egu), *Musa acuminata* (Mac), *Ananas comosus* (Aco), *Brachypodium distachyon* (Bdi), and *Oryza sativa* (Osa).

To further analyze the evolutionary characteristics of *BPC*s, we conducted a comparative analysis of the number, classes, and syntenic regions of *BPC*s across 15 species (**Figure 3**). These species include a basal extant flowering plant *A. trichopoda* (Atr), six dicot species, and eight monocot species, including coconut. The BPC gene members from 14 other plant species were detected using the same method described above for coconut and listed in **Supplementary Table S1**. *A. trichopoda* is one of the species with the fewest BPC genes, possessing only one member in Class I, which may represent the ancestral copy number state for this class. Among the other 14 species, the copy number of Class I genes has increased across the board. In dicot species, the Class I genes range from two to three copies, while in monocots, they range from two to eight copies. The palm species, such as *E. guineensis* (Egu) and *P. dactylifera* (Pda), exhibit four gene members. *M. acuminata* (Mac) having the top number (8) may result from the two WGDs (denoted as α and β) (D’Hont et al., 2012). Syntenic region comparisons indicated that these Class I *BPC* genes all originated from the same homologous chromosomal segment. This finding aligns with our previous analysis of the triplication of Class I genes in coconut and their syntenic relationship with BPCs in *Arabidopsis*.

For Class II, most of the analyzed species possess two or more *BPC* members, with the exception of *O. sativa* (Osa) and *B. distachyon* (Bdi) (**Figure 3**). A maximum of four BPC members were observed in species such as the dicots *M. domestica* (Mdo) and *C. sinensis* (Csi), as well as in the monocots *E. guineensis* (Egu) and coconut (Cnu). *A. trichopoda* has two genes belonging to Class II, but no syntenic chromosomal segments with *D. carota* (Dca) were detected. In the remaining species, Class II genes are located within syntenic regions, and different gene members are also found in homologous genomic regions. Among the six dicot species and the eight monocot species, excluding Osa and Bdi, the chromosomal regions containing BPCs exhibit strong synteny; however, no homologous segments were detected between dicots and monocots.

For Class III, *A. trichopoda*, along with four dicots (Dca, Vvi, Mdo, and Csi) and two monocots (Osa and Bdi), lacks a corresponding member. Additionally, there is only one gene present in Class III (**Figure 3**). Evolutionary analysis suggests that Class III originated from Class I or Class II members (Theune et al., 2019). Comparisons between coconut and *Arabidopsis*, along with the detection of conserved syntenic regions among monocots such as three species in the Palmae family (Egu, Pda, and Cnu) and *D. alata* (Dal) indicate that the Class III member originated from a common ancestor.

### CnBPC proteins were localized to the nucleus

**Figure 4:**
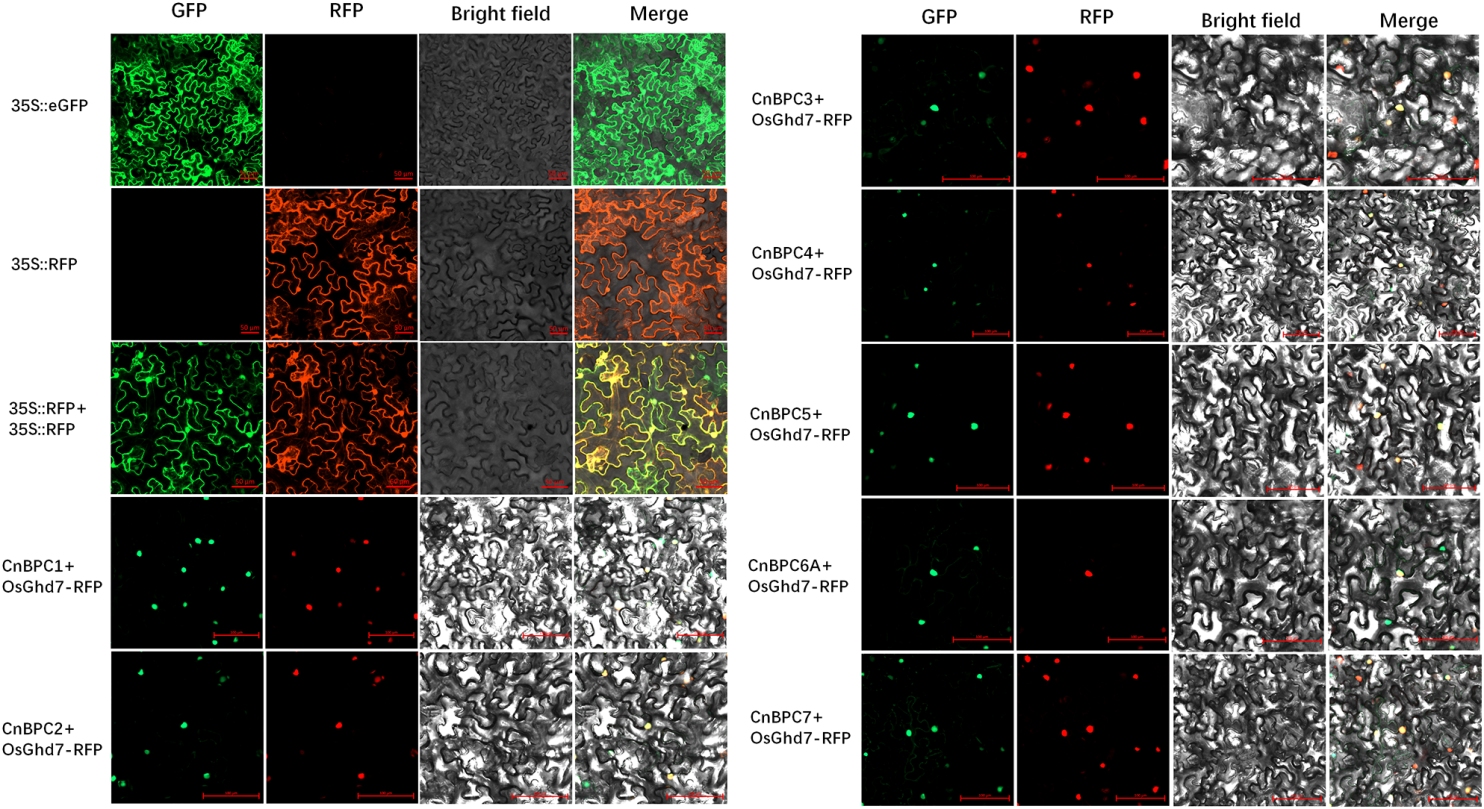
CnBPCs are localized to the nucleus. The 35S::CnBPC_(1-7)_: eGFP and nucleus marker 35S::OsGh7:RFP fusion protein were transiently expressed in tobacco epidermal cells. GFP signals were detected with time intervals between 48 h and 72 h post-infiltration using a confocal microscope (LMS980, Zeiss). GFP, green fluorescent protein; RFP, red fluorescent protein; Bright field, visible light; Merge, visible light merged with fluorescence. Scale bars, 50 μm or 100 μm.

BPC proteins were thought to be transcription factors that function primarily in the nucleus. Based on the NLS Mapper analysis, the CnBPC proteins were found to contain a nuclear localization signal sequence (NLS): PKPKKLKKV for Class I and either RKTKRMRKE or GRKAKRPRKE for Class II. However, no conserved NLS was detected for Class III. The subcellular localizations of *CnBPC*s were determined using transiently expressed CnBPC_(1-7)_-GFP fusion proteins in tobacco epidermal cells. The GFP signals were compared with the previously reported nucleus—localized OsGhd7-RFP. Fluorescence signals for the seven CnBPC_(1-7)_-GFP were observed in the nucleus (**Figure 4**). *CnBPC6B* had a low expression level and was not cloned in this study. The localization results also indicated that the *CnBPC*s show conservation for subcellular localization.

### Most genes in coconut have a GA motif in their promoter regions

**Figure 5:**
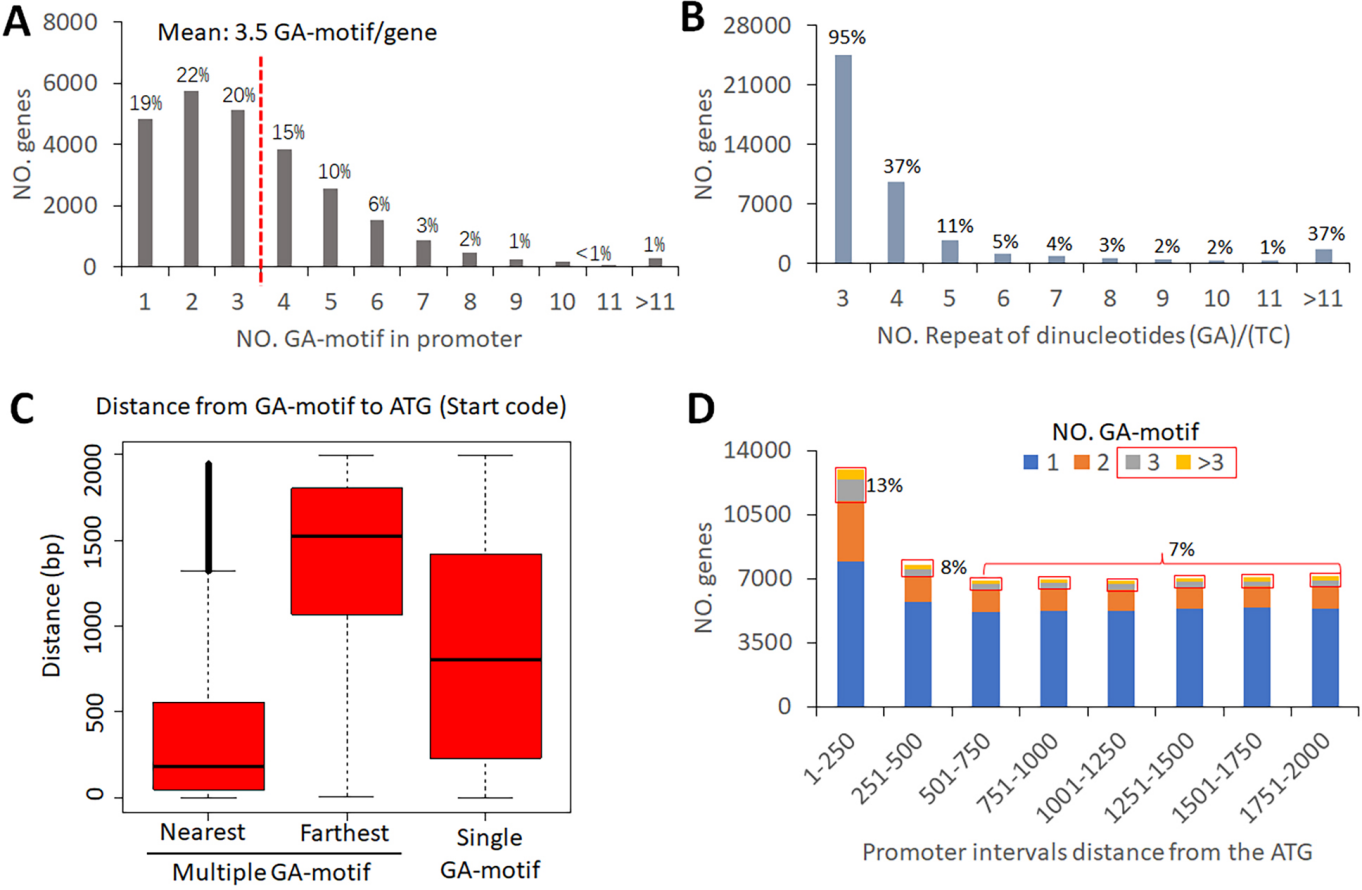
Features of GA/TC dinucleotide repeats in the promoters of coconut genes. **(A)** Distribution of NO. GA motif in the promoters. **(B)** Gene number with different GA/TC repeat times. **(C)** Quarterback diagram of GA motif’s distance from ATG. **(D)** Gene number with GA motifs in different regions of the promoter.

The *BPC* genes are known to bind with dinucleotide GA repeats (GA motif). In order to screen for potential *CnBPC* target genes, the 2-kb upstream sequence starting from the gene start codon for all coconut protein-coding genes (28,114) was extracted. Analyzing the 2-kb sequences indicated that approximately 92% (25,830) of genes were detected to have GA repeats with three or more repeat times, with an average of 3.5 GA motifs per gene (**Figure 5A**). The gene promoters with one to three GA motifs account for 51% of GA motif genes, while the rest could be considered GA-rich with four or more GA motifs in promoters. Among GA motifs, the most dominant type is the (GA)_3_ motif, which existed in 95% of GA motif-containing genes. The (GA)_4_ motif was found in 37% of GA motif genes. With the increase in GA repeat times, the proportion of occurrence in genes quickly decreased (**Figure 5B**).

The box plot shows that the distance between the GA motif and the gene start code is random for genes with a single GA motif, with the distance distribution slightly skewed toward 1,000 bp (**Figure 5C**). However, for genes with multiple GA motifs, 75% of GA motifs are within 500 bp from the start code for the nearest GA motifs, while 75% of GA motifs are more than 1,000 bp away. Further analysis of the distribution of GA motifs across the 2-kb promoter sequence revealed that the closest interval (1–250 bp) had the highest number of GA motif genes, and the remaining intervals had a similar number of genes (**Figure 5D**). Among all 250-bp intervals, a GA motif number higher than 2 was considered to indicate high GA motif density, accounting for 13% of genes in the 1–250-bp interval and 7%–8% of genes in the remaining intervals.

### *CnBPC* members interacted with gene promoters containing GA motifs in a different manner

**Figure 6:**
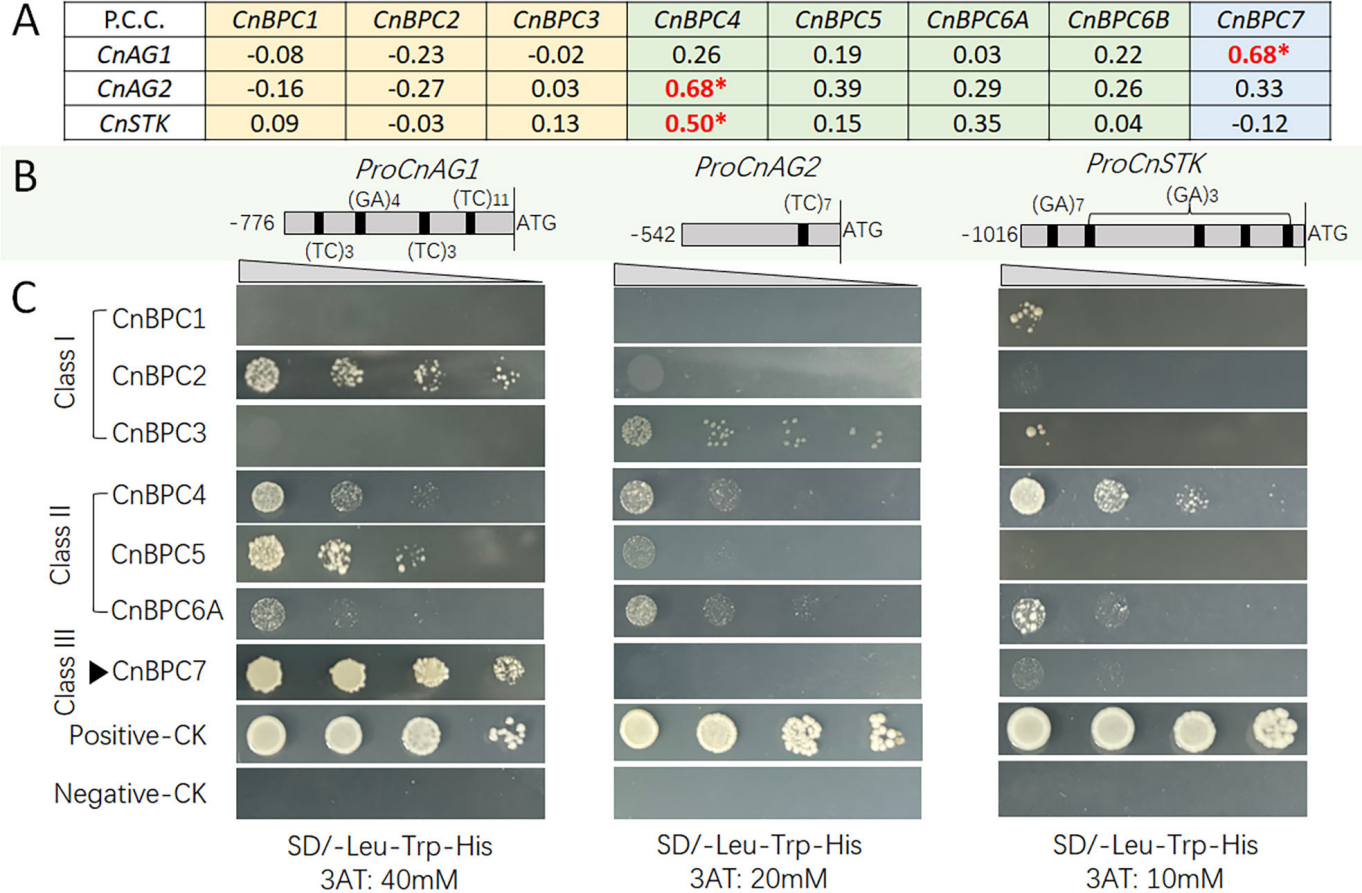
Correlation of *CnGA*/*CnSTK* expression with *CnBPC*s **(A)**, diagram of GA motifs in the promoters of *CnGA*/*CnSTK* **(B)**, and yeast one-hybrid analysis of interactions between *CnGA*/*CnSTK* promoters and *CnBPC*s **(C)**. Pearson’s correlation coefficient (PCC) was calculated based on the coconut transcriptomes used in this study, and the significance test was performed using *t*-test (cor.test in R, *p < 0.05). *CnAG1* and *CnAG2* are homologous genes of *AtAG* (AT4G18960), and *CnSTK* is homologous to *AtSTK* (AT4G09960). Positive-CK is the Y187 strain transformed with pGAD53m and pHIS2-p53. Negative-CK is the Y187 strain transformed with pGADT7 and pHIS2 empty vectors.

According to previous research, different plant species had shown varying tendencies to bind specific GA motifs and GA-rich regions attracting the binding of *BPC* genes. To validate and analyze the binding characteristics of *CnBPC*s, we chose a set of genes with a single GA motif, multiple GA motifs, and promoter sequences with different lengths of GA repeats for binding analysis. The MADS-box gene *AtSTK* was considered a putative target of *BPC*s. We selected three homologous genes—*CnGA1*, *CnGA2*, and *CnSTK*—that belong to the CD class of the ABCDE module responsible for regulating flower development in order to validate their interaction. The gene expression analysis indicated that *CnAG1* exhibited a positive correlation with *CnBPC7* (p < 0.05, **Figure 6A**). Additionally, *CnBPC4* had a positive correlation with *CnAG2* and *CnSTK*. Furthermore, the promoter region of three genes was also detected to have four, one, and five GA motifs, including (TC)_3_, (GA)_4_, (TC/GA)_7_, and (TC)_11_ (**Figure 6B**).

The Y1H assay between the *CnAG1* promoter and *CnBPC*s demonstrated that the CnBPC7 protein had stronger interaction with the *CnAG1* promoter than other *CnBPC* members, which was consistent with the high expression correlation between the two genes (**Figure 6C**). Meanwhile, the *CnAG1* promoter also had slightly weaker interactions with CnBPC2, CnBPC4, and CnBPC5 proteins. *CnAG2* had only one (TC)_7_ motif in its promoter, and all *CnBPC*s showed weak or undetectable interaction with this promoter fragment (−542 to −1). Additionally, the BPC Class II member *CnBPC4* interacted with the promoter of *CnSTK*, which had five GA motifs. This was consistent with the results of *AtSTK* regulation, whose promoter was bound by *BPC* Class I or Class II formed complex (Kooiker et al., 2005; Petrella et al., 2020).

**Figure 7:**
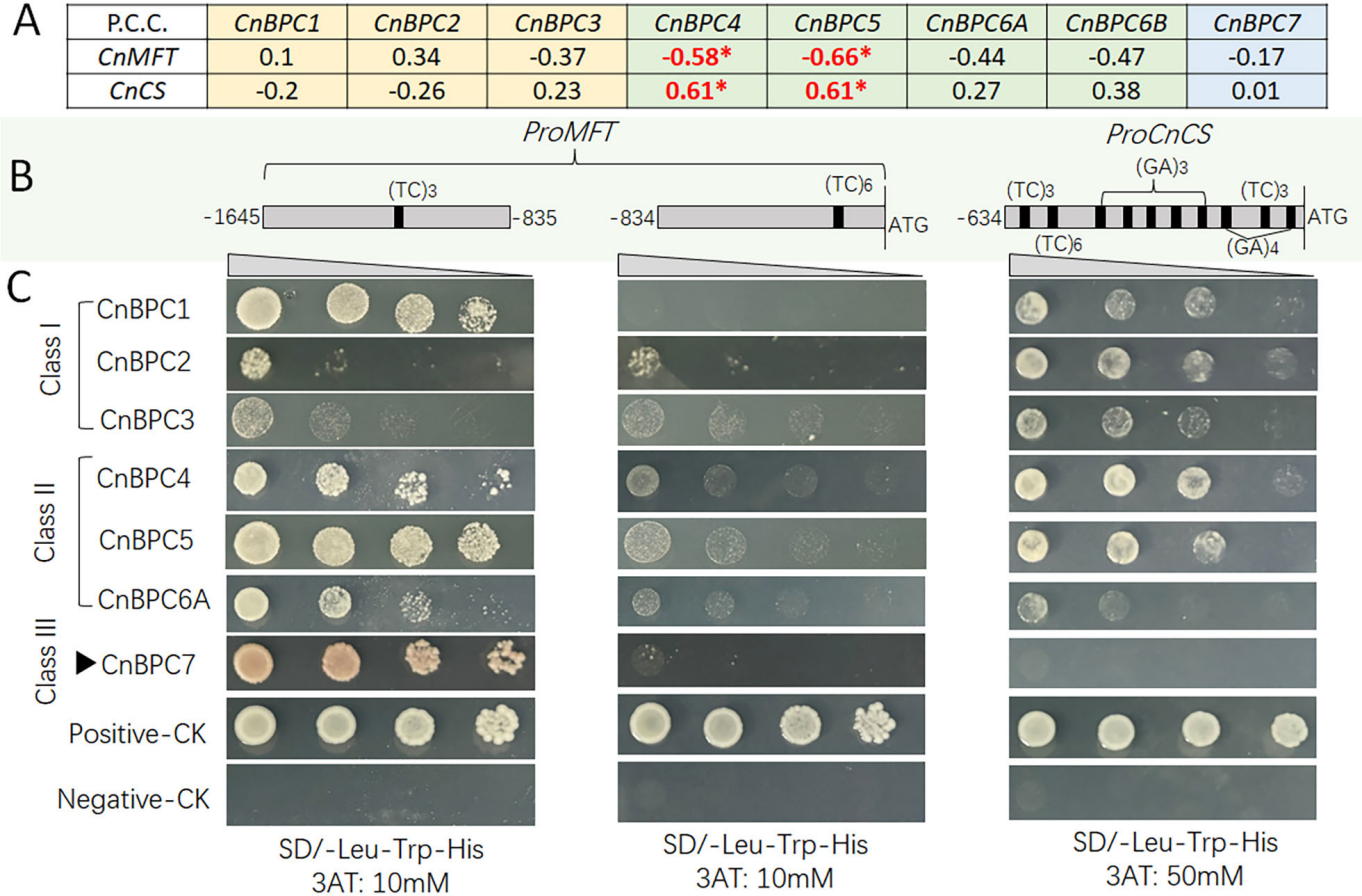
Correlation of *CnMFT*/*CnCS* expression with *CnBPC*s **(A)**, diagram of GA motifs in the promoters of *CnMFT*/*CnCS* **(B)**, and yeast one-hybrid analysis of interactions between *CnMFT*/*CnCS* promoters and *CnBPC*s **(C)**. Pearson’s correlation coefficient (PCC) was calculated, and the significance was tested the same as in **Figure 6**. *CnMFT* is a homologous gene of *AtMFT* (AT1G18100), and *CnCS* is homologous to *AtCESA6* (AT5G64740). Positive-CK is the Y187 strain transformed with pGAD53m and pHIS2-p53. Negative-CK is the Y187 strain transformed with pGADT7 and pHIS2 empty vectors.

In addition to the above three flower development-related genes, two other gene promoters were validated by the Y1H assay for interaction with the GA motif. *CnMFT* was selected because, in our Y1H coconut library screening with the *CnMFT* promoter, we identified *CnBPC1* and *CnBPC5* as candidates for promoter binding (data not shown). The expression pattern of *CnMFT* is also significantly negatively correlated with the expression levels of *CnBPC4* and *CnBPC5* (**Figure 7A**). Additionally, the promoter of *CnMFT* also contains GA motifs (TC)_3_ and (TC)_6_ (**Figure 7B**). *CnCS* was also selected for GA-motif interaction validation because it is positively co-expressed with *CnBPC4* and *CnBPC5* (**Figure 7A**) and has rich GA motifs—10 GA motifs— in the 600-bp promoter region adjacent to the start code (**Figure 7B**).

Since *CnMFT* contains two GA motifs in the promoter, the promoter was divided into two segments for the Y1H assay: CnMFT-F2 (−1,645 to −835) and CnMFT-F1 (−834 to −1) (**Figure 7B**). CnMFT-F2 contains one (TC)_3_ motif, and the Y1H assay indicated that it could have a stronger interaction with *CnBPC1*/*4*/*5*/*7* than with *CnBPC6A* (**Figure 7C**). Meanwhile, it was observed that CnMFT-F1 exhibited very weak interaction or undetectable interaction, indicating that the (TC)_6_ motif had weaker or no interaction with CnBPC proteins when compared with the (TC)_3_ motif. In contrast, *CnCS*, with its abundant GA motif, showed strong interaction with nearly all *CnBPC*s except for *CnBPC7*.

### Some *CnBPC*s could interact with specific *CnBPC* members

In the study of *AtBPC*s, it was found that *AtBPC*s could interact with other members within their subclass and are involved in gene regulation as part of the BPC complex (Simonini et al., 2012). We selected one representative from each BPC class: *CnBPC1*, *CnBPC5*, and *CnBPC7*. The yeast two-hybrid assay revealed that the CnBPC1 protein interacted with CnBPC6A, while CnBPC5 interacted with itself; however, the other gene pairs did not show any protein interactions in yeast (**Supplementary Figure S1**).

### Different *CnBPC* members tend to have different transcriptional activation effects on GA motifs

**Figure 8:**
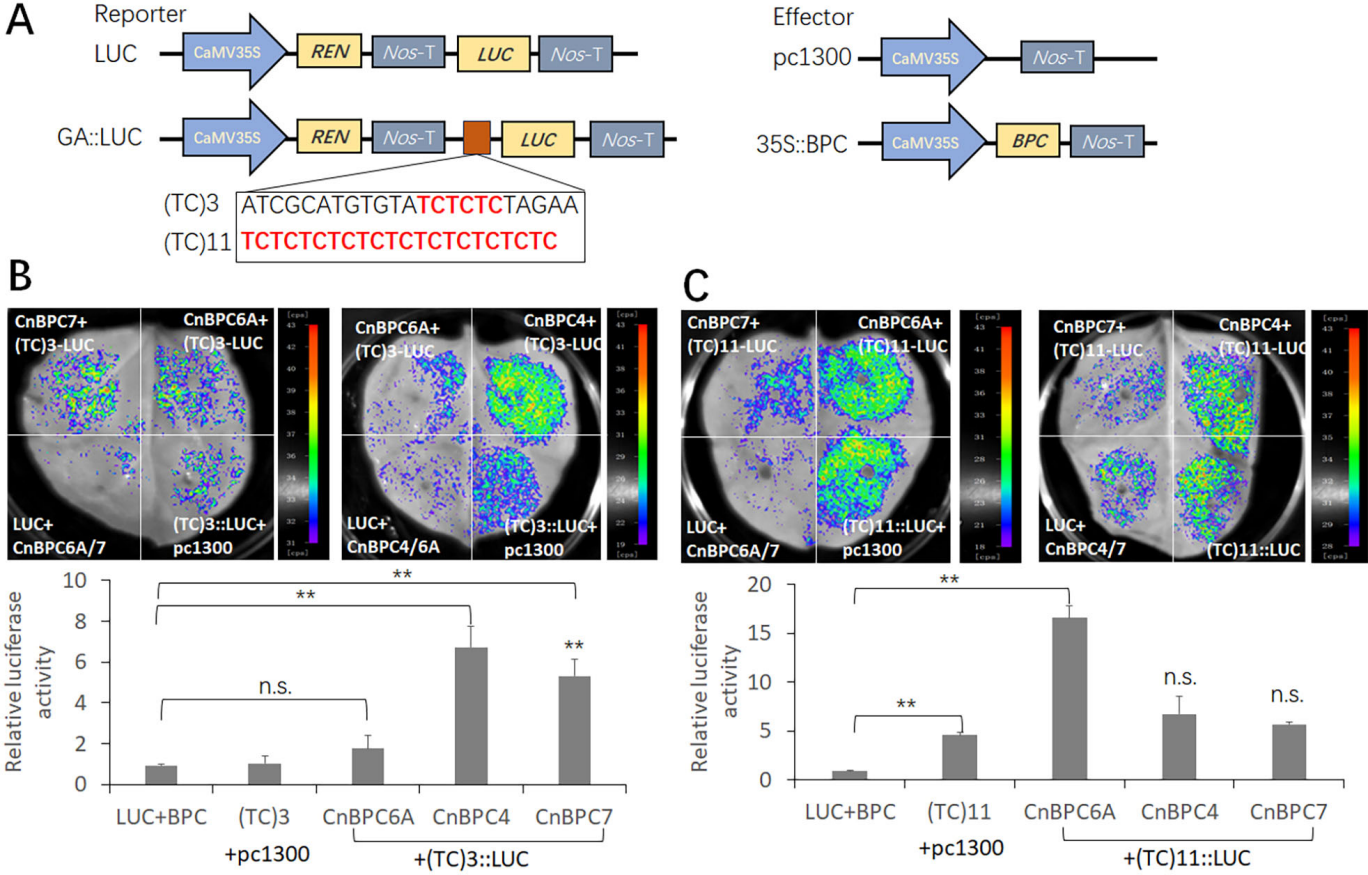
Transcriptional activation activity of CnBPC4, CnBPC6A, and CnBPC7 proteins in tobacco leaves. **(A)** The diagram of vectors used in dual-luciferase reporter assay. The coding sequence (CDS) of *CnBPC4*, *CnBPC6A*, and *CnBPC7* was cloned into the pc1300-35S vector to generate the effector. The sequence containing GA-motif - (TC)_3_ and (TC)_11_ were linked to the double-reporter vector *pGreenII 0800-LUC*. **(B)** LUC luminescence image and relative luciferase activity between GA-motif-(TC)_3_ and *CnBPCs*; and **(C)** between GA-motif-(TC)_11_ and CnBPCs. The vectors carrying the effectors were co co-transformed into *Nicotiana benthamiana* leaves along with the vectors carrying the reporter LUC. Three biological replicates were performed, the samples from three plants were as one replicate, and the error bars represent SE. Each value represents the means of six biological replicates. **P < 0.01 (Student’s t-test). The comparison was made between (TC)_3/11_-LUC + pc1300-35S and (TC)_3/11_-LUC+pc1300-35S-CnBPC.

According to the Y1H assay, CnBPCs could bind to DNA fragments with either one (GA/TC)_3_ motif or multiple GA motifs while exhibiting weak or negligible interactions with (TC)_6_ or (TC)_7_. We used a dual-luciferase reporter assay to further analyze the transcriptional regulation effects of these *CnBPC*s on the GA motif. Since *BPC*s in some species have been shown to interact with long GA tracts (Santi et al., 2003; Gong et al., 2018), we also selected the (TC)_11_ motif present in the CnAG1 promoter for the LUC assay, along with the (TC)_3_ motif (**Figure 8A**).

*CnBPC4*, *CnBPC6A*, and *CnBPC7* were chosen for analysis since strong interactions were detected in the Y1H assay. The dual-LUC assay showed that the (TC)_3_ motif had no effects on activating the reporter gene, but the (TC)_11_ motif showed a significant increase in the luciferase gene transcription (**Figures 8B, C**). *CnBPC4* and *CnBPC7* had significant transcriptional activation of the (TC)_3_ motif, while no significant transcriptional activation activity was detected for *CnBPC6A* (**Figure 8B**). For the (TC)_11_ motif, only *CnBPC6A* exhibited a significant transcriptional activation effect for the motif when compared to both control vectors. However, *CnBPC4* and *CnBPC7* only exhibited luciferase activity compared to that of the (TC)_11_-LUC vector, indicating that they had no significant effect on the transcriptional levels of the (TC)_11_ motif.

According to the Y1H assay, the promoter containing a single (TC)_3_ motif could interact with nearly all *CnBPC*s, although the interaction intensities varied (**Figure 7C**). *CnBPC1* and *CnBPC2* from Class I, along with *CnBPC5* from Class II, were selected for the LUC assay (**Supplementary Figure S2**). The LUC assay indicated that the *CnBPC1* and *CnBPC5*, which demonstrated strong interactions in the Y1H assay, had significant transcriptional repression of the (TC)_3_ motif. In contrast, no significant transcriptional activation activity was detected for *CnBPC2*, which had weak interaction with the promoter sequence MFT-F2 containing the (TC)_3_ motif in the Y1H assay.

### The expression profiles of *CnBPC*s and predictions of their target genes

The Y1H and dual-LUC assays mentioned above showed that the (GA/TC)_3_ motif is commonly bound by *CnBPC*s, and promoters rich in GA motifs are highly likely to be regulated by *CnBPC*s (**Figure 7**). In addition, genes having significant expression correlation with *CnBPC*s had an extra-high possibility of being the targets of *CnBPC*s (**Figures 6**, **7**). To identify the most probable targets for *CnBPC*s, we analyzed the expression patterns and co-expression modules of *CnBPC*s and GA motif-containing genes.

**Figure 9:**
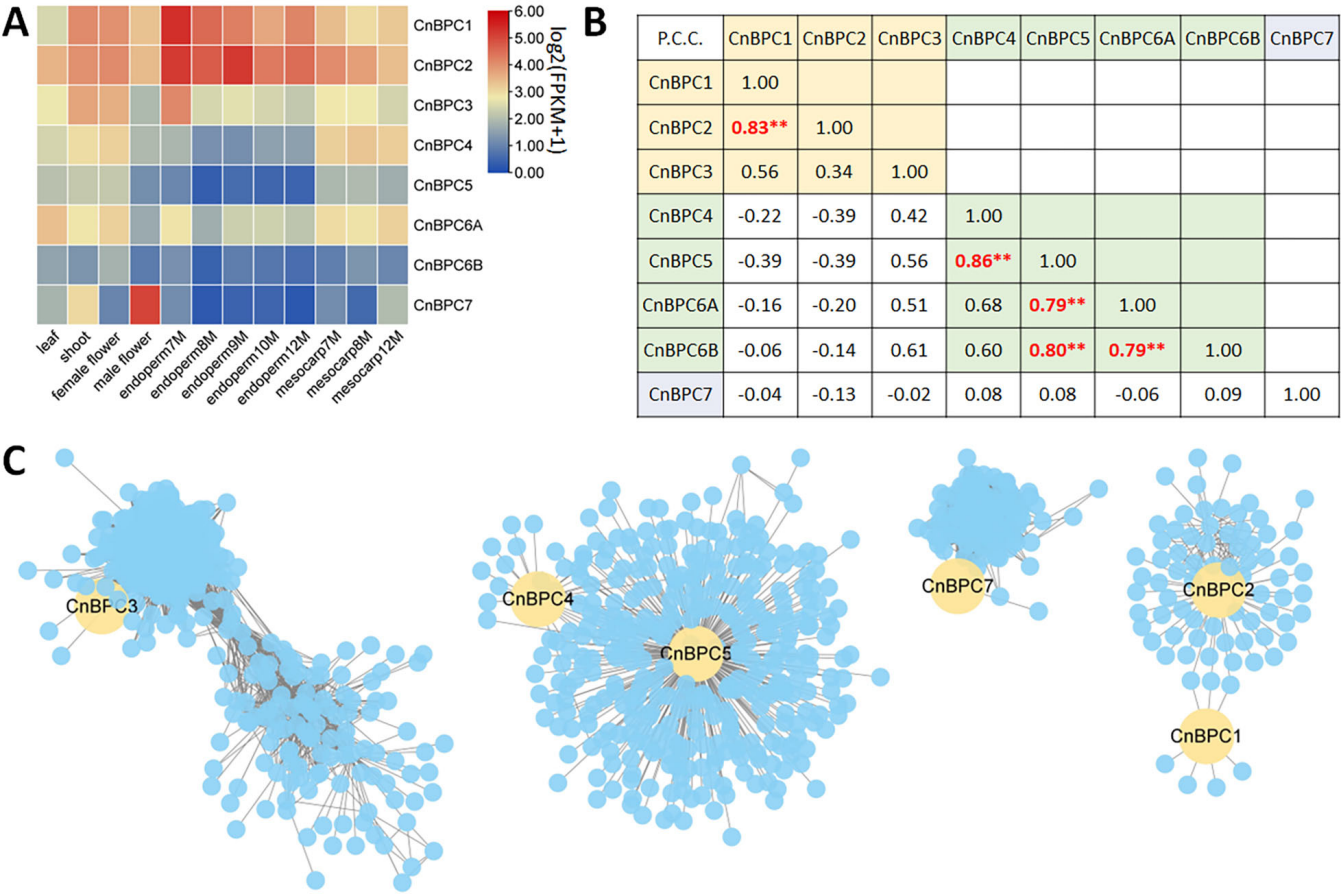
The expression pattern of *CnBPC*s and co-expression network between *CnBPC*s and GA motif-containing genes. **(A)** The *CnBPC* expression heatmap-based log2-transformed mean FPKM for coconut leaf, shoot, endosperm, and mesocarp tissues, which is based on the transcriptomes used in this study. **(B)** *CnBPC* expression correlation. Pearson’s correlation coefficient (PCC) between *CnBPC*s was calculated based on the coconut transcriptomes, and the significance test was performed using *t*-test (cor.test in R, **p < 0.01). **(C)** The co-expression network for *CnBPC*s and GA motif-containing genes generated by Weighted Gene Co-Expression Network Analysis (WGCNA). The co-expression network was constructed including all genes expressed with FPKM value higher than one in at least one transcriptome and the transcriptome used in this study.

Based on the transcriptomes from five kinds of coconut tissues—leaf, stem, flower, endosperm, and mesocarp—the expression of different *CnBPC*s tended to have the same expression pattern within the class (**Figures 9A, B**). For Class I, *CnBPC1* and *CnBPC2* had a high positive co-expression correlation and were expressed in all coconut tissues with high expression levels in the endosperm. *CnBPC4* to *CnBPC6* had relatively low expression in the endosperm but relatively high expression in the mesocarp. This class also has a significant positive expression correlation with *CnCS* (PCC, 0.61), which was supposed to be related to cellulose synthesis in coconut mesocarp (**Figure 7**).

To systematically identify gene correlation with *CnBPC*s in expression levels, 18,615 genes (FPKMmax > 1) were used for co-expression network construction. Four modules containing *CnBPC*s were found, with 426 GA motif genes (**Figure 9C**, **Supplementary Table S3**). The co-expression modules also showed that *CnBPC*s from the same class were located within the same module: *CnBPC4* and *CnBPC5*, *CnBPC1* and *CnBPC2*. The Y1H-validated *CnC*S was also detected within the same co-expression module with *CnBPC4* and *CnBPC5* (**Supplementary Table S3**). These GA motif genes could be candidates for being regulated by *CnBPC*s.

## Discussion

*BPC*s play a critical role in various plant growth and developmental responses. A dysfunction of BPCs can lead to a range of pleiotropic developmental defects. Additionally, *BPC*s have five highly conserved cysteine residues in the basic zinc finger-like DNA-binding domain and very conserved target sequences, specifically the GA motif among plant species. Therefore, it offers a clear basis for predicting and validating their targets. However, GA motif-bearing genes are widespread throughout genomes, making it important to identify the most likely target genes. Coconuts are important crops and landscape plants in tropical areas and are characterized by their large fruits and liquid endosperm known as coconut water. However, little is known about the types of GA motifs that *BPC* genes in coconuts would bind to and whether they play a role in coconut fruit development.

The ability of BPC proteins to bind to GA motifs is a conserved function observed in both plants and animals (Berger and Dubreucq, 2012; Rieder et al., 2017). Furthermore, many studies have shown that *BPC* genes have function redundancy (Monfared et al., 2011; Shanks et al., 2018; Wu et al., 2020). Evolutionary analysis of *BPC* in this study revealed that these genes are highly conserved among different plant species, exhibiting a remarkable degree of synteny in the chromosomal regions that contain these genes across various species. Given that *BPC*s retain a conserved DNA-binding domain (Theune et al., 2019), it is likely that the origin of *BPC* members through segmental duplication or whole-genome duplication contributes to their functional redundancy. *BPC*s within the same class are highly conserved in both protein sequences and DNA-binding motifs, which contributes to functional conservation to a certain extent (Simonini et al., 2012; Simonini and Kater, 2014; Petrella et al., 2020). *BPC*s can regulate the expression of target genes by binding to GA motifs (Wu et al., 2020), suggesting that the expression patterns of *BPC*s may influence their spatiotemporal regulation. Our research indicated that the expression patterns of *CnBPC*s within the same class are also highly similar, ensuring that their functional roles overlap in both space and time.

Although the binding motifs of *BPC*s are conserved, the binding preference for GA motifs varies slightly among different species, which may be related to the divergence of the conserved DNA-binding domains (Kooiker et al., 2005; Berger et al., 2011; Berger and Dubreucq, 2012). Furthermore, in addition to the functional redundancy of *BPC*s, different *BPC* members within the same species can exhibit divergent functions. For instance, in rice, *OsGBP1* represses grain length and seedling growth, while *OsGBP3* promotes grain length and plant height (Gong et al., 2018). The BPC genes transactivate genes containing the GA motif, such as *BBR* in barley (Santi et al., 2003) and *LEC2* in *Arabidopsis* (Berger et al., 2011), repress target genes (Mu et al., 2017), or exhibit exquisite epigenetic control by orchestration of the deposition of H3K27me3 on the GA motif (Hecker et al., 2015; Petrella et al., 2020). Y1H and dual-LUC assay suggested that *CnBPC*s interacted with the GA motif with gene bias, and genes with significant expression correlations had stronger interaction with *CnBPC*s. The transcriptional activation and repression activities of *CnBPC*s, along with their co-expression with target genes, suggest that different *CnBPC*s could have distinct gene functions. The regulatory mechanisms of *BPC*s are further complicated by their interactions with other transcription factors and chromatin remodeling proteins (Li et al., 2023; Sahu et al., 2023; Zhao et al., 2024). This also provides a basis for further research into the gene functions of *BPC*s and for elucidating their molecular mechanisms of action, which can begin by screening for interacting genes associated with *BPC*s.

*BPC*s are involved in the regulation of plant growth and development, particularly in plant height, roots, flowers, and seeds (Ma et al., 2022; Feng et al., 2023; Dang et al., 2024; Zhao et al., 2024). Due to the widespread presence of GA motifs in gene regions, *BPC*s have the potential to regulate many genes. In coconut, 92% of protein-coding genes had at least one GA motif in the promoter, and (GA)_3_ is the most widespread motif type. This could be explained by the research indicating that the GA motif was one core group around transcriptional start sites (Yamamoto et al., 2009). Y1H suggested that promoters with multiple GA motifs could be regulated by more *CnBPC*s. Research on the GA-rich promoter of *AtSTK* indicated that other *BPC* Class I and Class II factors could act redundantly in the regulation of *AtSTK* (Simonini et al., 2012; Petrella et al., 2020). These results suggested that the redundancy in *BPC* function could occur in genes with a high abundance of GA motifs in their promoters. Additionally, the similar expression patterns of *BPC* members support the occurrence of functional redundancy.

Our research validated the interaction between CnBPC proteins and the promoters of *CnAG1*, *CnAG2*, *CnSTK*, *CnMFT*, and *CnCS*, suggesting their involvement in seed germination, flower development, and mesocarp development. Through the construction of a co-expression network and analysis of GA motif features, we identified 426 genes as potential targets of *CnBPC*s. This work provides a foundation for exploring the potential target genes of *CnBPC*s and lays a theoretical groundwork for analyzing the role of *CnBPC*s in coconut flower and seed development.

## Data Availability

*CnMFT* (AZ15G0262410), *CnAG1* (AZ01G0023710), *CnAG2* (AZ04G0092990), *CnSTK* (AZ12G0230100), *CnCS* (AZ07G0160020), and coconut RNA-seq SRAs (accession number CRA004778) are publicly accessible at https://ngdc.cncb.ac.cn/gsa.

## Supplementary material

Supplementary Figure S1 Yeast Two-Hybrid Analysis of CnBPC1, CnBPC5, and CnBPC7 interactions with all CnBPC Proteins. *CnBPC1/5/7* were cloned to pGBKT7 vector and fused with GAL4 DNA-binding domain as baits, while all *CnBPCs* were cloned to pGADT7 vector and fused with GAL4 activation domain as preys. Different combinations of plasmids for bait and prey were co-transformed in AH109 yeast strain. p53 and SV40 large T-antigen are two proteins that are known to interact in yeast were used as a positive control. LaminC and SV40 large T-antigen were used as a negative control.

Supplementary Figure S2 Transcriptional repression activity of CnBPC1, CnBPC2, and CnBPC5 proteins in tobacco leaves. **(A)** The diagram of vectors used in dual-luciferase reporter assay. **(B)** Relative luciferase activity of the dual-luciferase reporter assay. The methods were same as in **Figure 8**.
