# Supplementary figures and images for: Genome-wide identification and characterization of BASIC PENTACYSTEINE transcription factors and their binding motifs in coconut palm
Source: Front Plant Sci. 2024 Dec 10;15:1491139. doi: 10.3389/fpls.2024.1491139 (PMC11666369; doi:10.3389/fpls.2024.1491139)

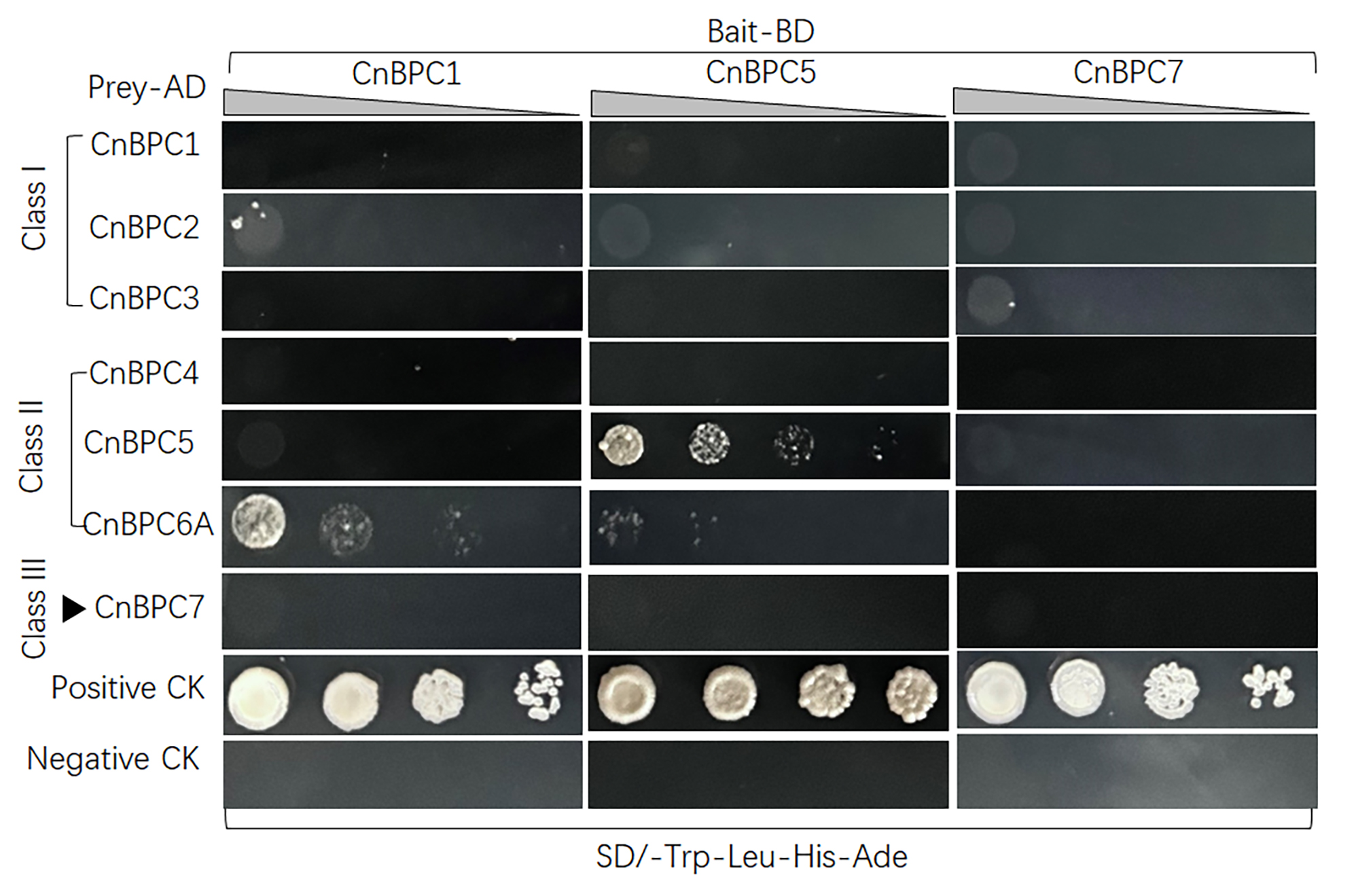

Supplement: Supplementary Figure S1 — Yeast Two-Hybrid Analysis of CnBPC1, CnBPC5, and CnBPC7 interactions with all CnBPC Proteins. CnBPC1/5/7 were cloned to pGBKT7 vector and fused with GAL4 DNA-binding domain as baits, while all CnBPCs were cloned to pGADT7 vector and fused with GAL4 activation domain as preys. Different combinations of plasmids for bait and prey were co-transformed in AH109 yeast strain. p53 and SV40 large T-antigen are two proteins that are known to interact in yeast were used as a positive control. LaminC and SV40 large T-antigen were used as a negative control. [file Image1.jpeg]

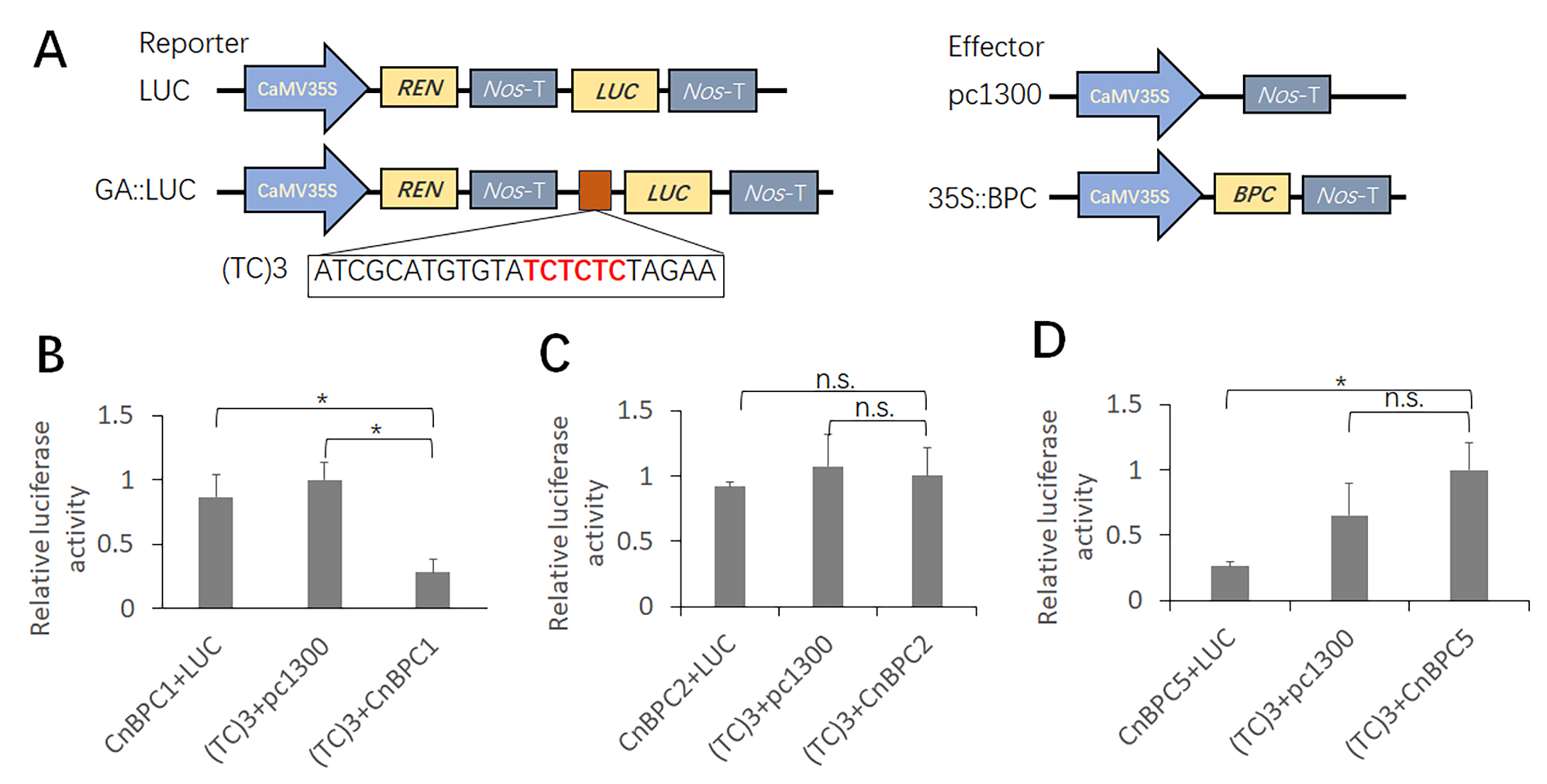

Supplement: Supplementary Figure S2 — Transcriptional repression activity of CnBPC1, CnBPC2, and CnBPC5 proteins in tobacco leaves. (A) The diagram of vectors used in dual-luciferase reporter assay. (B) Relative luciferase activity of the dual-luciferase reporter assay. The methods were same as in Figure 8 . [file Image2.jpeg]
